# Mn(III) Porphyrin MnTE-2-PyP^5+^ Associated with Ascorbate: A Redox-Active Therapeutic Strategy against Leishmaniasis

**DOI:** 10.1021/acsinfecdis.5c00520

**Published:** 2025-10-10

**Authors:** Tiago H. S. Souza, Jacqueline C. Bueno-Janice, Letícia S. Vasconcelos, Paulo E. Cabral Filho, Julio S. Reboucas, Regina C. B. Q. Figueiredo, Adriana Fontes

**Affiliations:** † Departamento de Biofísica e Radiobiologia, 28116 Universidade Federal de Pernambuco (UFPE), Recife, Pernambuco 50670-901, Brazil; ‡ 92923 Instituto Aggeu Magalhães, Fundação Oswaldo Cruz (FIOCRUZ), Recife, Pernambuco 50740-465, Brazil; § Departamento de Química, Universidade Federal da Paraíba (UFPB), João Pessoa, Paraíba 58051-900, Brazil

**Keywords:** Leishmania spp., neglected disease, Mn(III) porphyrin, vitamin C

## Abstract

Toxicity and rising resistance to current leishmaniasis drugs highlight the need for alternative therapies. Manganese porphyrins (MnPs) have demonstrated therapeutic potential in various oxidative stress-based diseases/ailments due to their redox-modulating properties. Thus, this study aimed to evaluate the redox-active effects of MnTE-2-PyP^5+^ (BMX-010, AEOL10113, MnP ethyl) combined with ascorbate (Asc, vitamin C) on *Leishmania amazonensis*, *Leishmania braziliensis*, and *Leishmania chagasi* *in vitro*. The effects on promastigote growth were assessed, and the mechanism of action was studied by quantifying reactive oxygen species (ROS) and using catalase to evaluate H_2_O_2_ involvement. The effects on intracellular amastigotes and the mitochondrial membrane potential (ΔΨm) of promastigotes from the most susceptible species were evaluated. Cytotoxicity assays were carried out on mammalian cells. MnP ethyl alone had no impact on parasite growth; however, MnP ethyl/Asc treatment led to a significant reduction in the promastigote growth: 88% for *L. amazonensis*, 43% for *L. chagasi*, and 37% for *L. braziliensis* after 48 h. MnP ethyl/Asc generated about 300% more ROS than the untreated control and induced ΔΨm depolarization. Catalase addition restored parasite survival, confirming H_2_O_2_ as the primary mediator of the MnP ethyl/Asc effect. Moreover, MnP ethyl/Asc exhibited minimal cytotoxicity on mammalian cells. The MnP ethyl/Asc treatment reduced the infection index by about 58% and the number of amastigotes per macrophage by 42% in *L. amazonensis* after 24 h. These findings demonstrated that MnP ethyl/Asc exerted an antileishmanial effect through oxidative stress, providing a promising alternative for preclinical evaluation.

## Graphical abstract

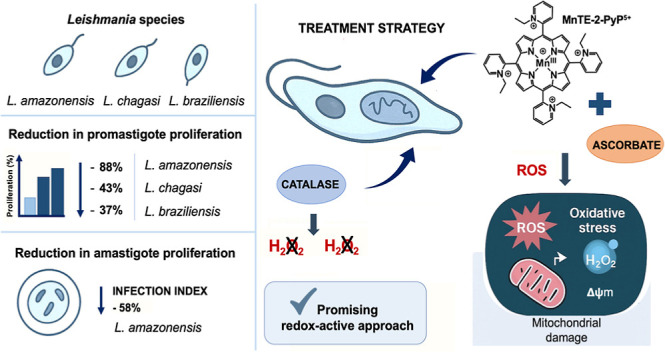

Leishmaniasis comprises a group of parasitic diseases caused by protozoa of the genus *Leishmania*. According to the World Health Organization (WHO), leishmaniasis is one of the most prevalent parasitic diseases globally and is classified as a neglected disease due to the limited investment in new therapeutic strategies, representing a significant global public health challenge. The disease presents three main forms: cutaneous (CL), mucocutaneous (MCL), and visceral leishmaniasis (VL, also known as kala-azar). CL is the most common form and can progress to secondary lesions, leading to diffuse cutaneous leishmaniasis. MCL is the most disabling form, while VL is the most severe. It is estimated that over 1 million new cases of CL each year, and 30,000 cases of VL occur worldwide. In the Americas, *Leishmania amazonensis* and *Leishmania braziliensis* are the primary species responsible for CL; *L. braziliensis* also causes MCL, and *L. amazonensis* is associated with diffuse cutaneous leishmaniasis. *Leishmania chagasi* is the species most frequently involved in VL.^1,2^

Currently, the drugs used to treat leishmaniasis are highly toxic and include compounds such as pentavalent antimonials (*e.g.*, meglumine antimoniate and sodium stibogluconate), amphotericin B, and miltefosine. Additionally, cases of parasite resistance to conventional treatments have been reported, which limits the use of these compounds.^3–5^ As a result, there is an urgent need to develop more effective and safer therapeutic alternatives. In this context, *in vitro* and *in vivo* studies have been conducted using water-soluble Zn­(II) porphyrins as photosensitizers in the photodynamic inactivation of *L. amazonensis* and *L. braziliensis*.^6–9^ Additionally, two Mn­(III) complexes of high lipophilicity, derived from neutral water-insoluble porphyrins, have also been reported for photodynamic inactivation of *L. braziliensis* and *Leishmania panamensis*.^10,11^ In a different approach, porphyrins complexed with metals such as antimony­(V) and bismuth­(III) have also been investigated as candidates for the treatment of leishmaniasis, demonstrating promising *in vitro* antileishmanial activity.^12^ These findings have prompted us to investigate water-soluble Mn­(III)-porphyrins (MnPs) as novel redox-active agents for leishmaniasis treatment, in a nonphotodynamic setting. To date, as far our knowledge goes, no studies have evaluated MnPs in a redox-active context with Asc against *Leishmania* spp..

MnPs have shown promise in *in vivo* studies, particularly in cancer treatment and as a radioprotective agent, due to their ability to function as either pro- or antioxidants, depending on the redox conditions of their microenvironment.^13,14^ When MnPs are combined with reducing agents like ascorbate (Asc, vitamin C), they exhibit pro-oxidant activity, catalyzing the autoxidation of Asc and generating H_2_O_2_.^15–18^ Asc has been selected as the MnP reducing agent due to its favorable safety profile at controlled dosages and widespread use in *in vivo* applications.^19–21^

Tovmasyan et al. (2015)^22^ and Ye et al. (2011)^16^ conducted independent studies to assess the anticancer potential of the MnP/Asc system, evaluating 14 different MnPs with varying redox properties, charges, sizes, and lipophilicity. In both studies, the Mn­(III) *meso*-tetrakis­(*N*-ethylpyridinium-2-yl)­porphyrin (MnTE-2-PyP^5+^, also known as BMX-010 and AEOL10113; shortened in this manuscript to MnP ethyl) was identified as the most effective catalyst for Asc autoxidation, demonstrating the greatest ability to induce cancer cell death. Like tumor cells, *Leishmania* spp. rely on a limited antioxidant system that can scavenge reactive oxygen species (ROS) only up to a certain threshold. Once this limit is exceeded, the parasites are unable to efficiently detoxify ROS, leading to oxidative damage and cell death.^23–25^ This vulnerability parallels that observed in cancer cells, where MnP/Asc-induced ROS production overwhelms the weakened antioxidant defenses. Based on these similarities, MnP ethyl was selected as a potential antileishmanial agent in the present study. Another aspect of note on MnP ethyl structural design is its permanent positive charge due to the *N*-alkylpyridinium groups, which can enhance its interaction with negatively charged parasite membranes.^26–28^

The biomimetic properties,^29–31^ chemical stability,^29,32,33^ well-defined pharmacokinetics,^34,35^ and safe toxicity profile of MnTE-2-PyP^5+^ in animal models and humans^13,36–38^ have made MnTE-2-PyP^5+^ a remarkable redox-active therapeutics and a lead compound in many exploratory nonclinical studies,^13,29,39–41^ translational medicine, and clinical trials.^36–38,42^ Indeed, MnTE-2-PyP^5+^ was well tolerated in phase I clinical trials^13,38^ and progressed to various phase II clinical trials on atopic dermatitis and itch.^37,38^

Thus, the aim of this study was to evaluate the *in vitro* activity of the MnP ethyl/Asc combined treatment against *Leishmania* spp. responsible for the three major forms of the disease (CL, MCL, and VL). This approach aims to identify alternatives to overcome the limitations of current leishmaniasis treatments, including their adverse effects and the emergence of resistance. Given the current status of MnTE-2-PyP^5+^ as a lead redox-active therapeutic agent and the promising results of the MnP/Asc combination in cancer therapy, this study explores, for the first time, the MnP ethyl/Asc system as a novel candidate for leishmaniasis treatment.

## Results

### The Association between MnP and Ascorbate Reduces *Leishmania* spp. Proliferation

For *L. amazonensis*, after 48 h, both combinations of 5 μM MnP ethyl and 3 mM Asc, and of 10 μM MnP ethyl and 2 mM Asc, were effective, inhibiting growth by more than 85% compared to the control (parasites in medium only). No significant difference was observed between the negative control and the treatment with MnP alone (10 μM). However, treatment with Asc alone at 3 mM reduced *L. amazonensis* proliferation. For *L. braziliensis* and *L. chagasi*, neither Asc (3 mM), MnP ethyl (10 μM), nor the control groups showed statistical differences. When treated with 5 μM MnP ethyl + 3 mM Asc combined treatment, growth inhibition reached 37% for *L. braziliensis* and 43% for *L. chagasi*, while the combination of 10 μM MnP ethyl + 2 mM Asc reduced growth by 32% and 33%, respectively, compared to the negative control (Figure 1). The inhibition after 24 h was similar to those observed at 48 h (Supporting Information, Figure S1). Further experiments were performed with *L. amazonensis* which showed to be more sensitive to the treatment.

**Figure 1:**
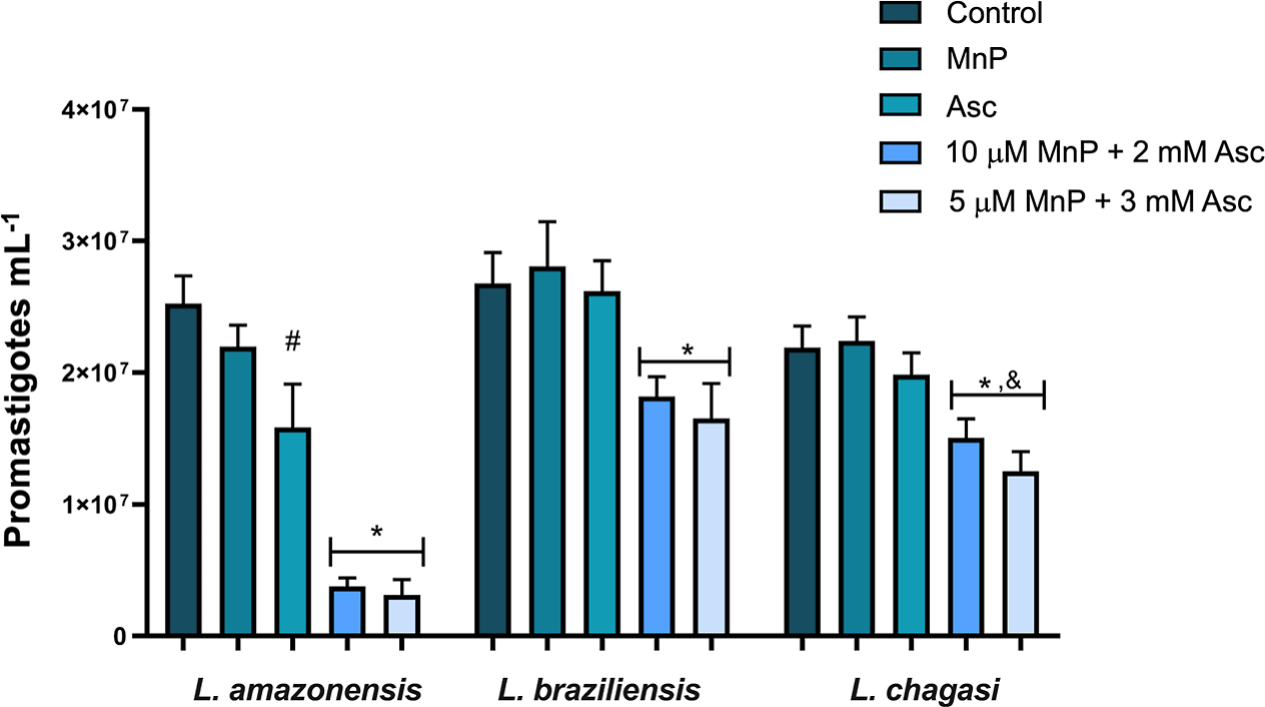
Effect of the treatments on the promastigote growth after 48 h of incubation. MnP: MnP ethyl (10 μM). The concentration of Asc alone was 3 mM. Data presented as mean ± standard deviation (SD). Significantly different (*p* < 0.05) from the control, MnP, and Asc groups*, between Asc and the control^#^, and between the MnP/Asc groups^&^. The experiments were conducted in duplicate in at least three independent assays.

### MnP/Asc Treatment Produces ROS

ROS production was assessed using the fluorescent probe 2′,7′-dichlorofluorescein diacetate (DCFH-DA) in *L. amazonensis*, which was chosen for its greater susceptibility to the treatment compared to other species. The fluorescence emitted is proportional to the level of ROS generated. As shown in Figure 2, the combined treatments with MnP ethyl/Asc (5 μM MnP ethyl + 3 mM Asc or 10 μM MnP ethyl + 2 mM Asc) resulted in over 300% higher fluorescence signals relative to the control, indicating a significant increase in ROS production. MnP ethyl alone caused only a slight increase in ROS, while the group treated with Asc alone showed a minor reduction in ROS production compared to the control.

**Figure 2:**
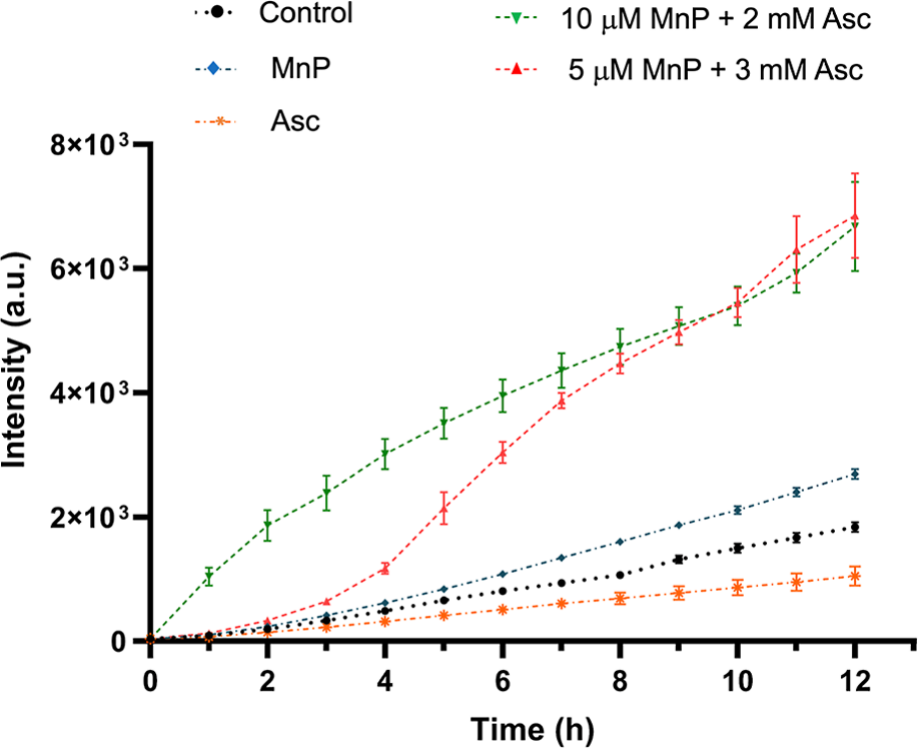
Progressive ROS generation over time visualized using the fluorescent probe DCFH-DA in *L. amazonensis* promastigotes. The concentrations of MnP ethyl alone and Asc alone were respectively 10 μM and 3 mM. MnP ethyl alone and Asc alone were used as controls at their respective highest concentrations applied in the promastigote assays. For the combined treatment, concentrations are given in the plot. Data are presented as mean ± standard error (SE). a.u. = arbitrary units. Experiments were performed in triplicate across two independent assays.

### Catalase Presence Reduces MnP/Asc Treatment Efficacy

The cytotoxicity of MnPs/Asc in cancer cells is largely attributed to the production and accumulation of H_2_O_2_ via MnP-catalyzed autoxidation of Asc; in these studies, the MnP/Asc effect is suppressed or decreased by treatment with catalase.^17,43,44^ To investigate whether a similar mechanism occurs in leishmaniasis after MnP ethyl/Asc treatment, catalase, an enzyme that efficiently converts H_2_O_2_ into water and oxygen, was added to the parasite cultures. This assay was performed with *L. amazonensis* promastigotes, which were identified as the most sensitive species to the treatment.

The addition of catalase significantly reduced the treatment efficacy, leading to increased parasite proliferation. For the combination of 5 μM MnP ethyl and 3 mM Asc, growth suppression dropped from 88% to just 14% in the presence of catalase. Similarly, for the 10 μM MnP ethyl + 2 mM Asc treatment, suppression decreased from 85% to 12% (Figure 3). The inhibition data after 24 h followed a similar trend to that observed at 48 h (Supporting Information, Figure S2).

**Figure 3:**
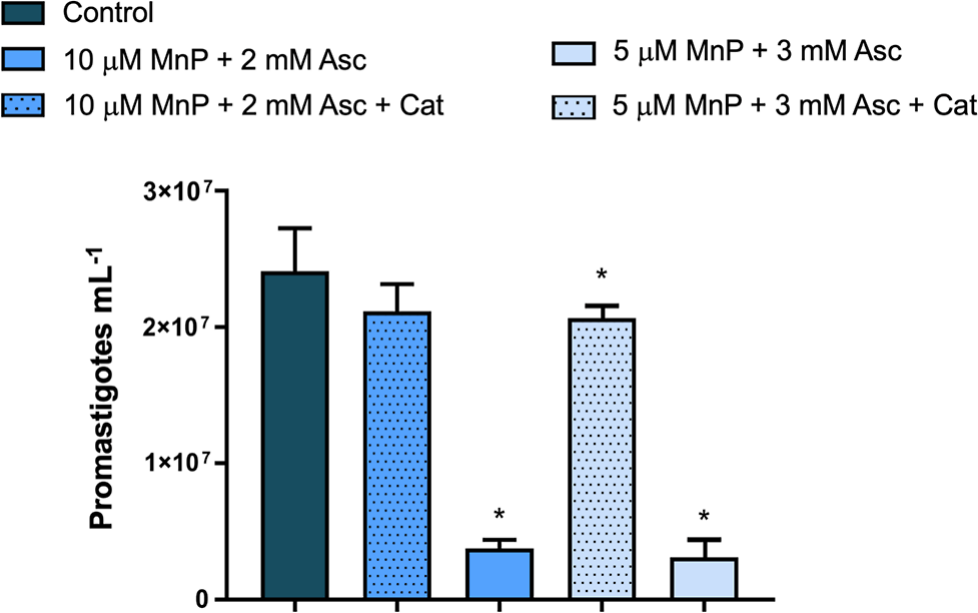
Reduction in the efficacy of MnP/Asc treatment on *L. amazonensis* promastigote growth after 48 h, due to the addition of catalase. Cat.: Catalase 500 to 1250 units mL^–1^. Data presented as mean ± standard deviation (SD). MnP: MnP ethyl. *Significantly different from the control (*p* < 0.05). Experiments were performed in triplicate in at least three independent assays.

### The Treatment MnP/Asc Reduces Mitochondrial Membrane Potential of Parasite

In this assay, to evaluate the effects of treatments on mitochondrial function of *L. amazonensis*, Rhodamine 123 (Rh 123), a fluorescent dye commonly used to monitor mitochondrial membrane potential (ΔΨm), was employed. A decrease in Rh 123 fluorescence intensity indicates ΔΨm depolarization, while an increase suggests hyperpolarization. Median fluorescence intensities (MFIs) were evaluated by flow cytometry.^45^

In the group treated with 3 mM Asc, a 34% reduction in MFI was observed (Figure 4), indicating mild ΔΨm depolarization, corroborated with the inhibition of cell proliferation. The MnP ethyl 30 μM group showed a 28% reduction in MFI, at a concentration three times higher than that used in the treatment. However, this reduction appears to be a transient effect, as no significant changes in *Leishmania* spp. proliferation were observed after 48 h compared to the control. Statistically significant differences were observed between the untreated control and both MnP ethyl and Asc single-treatment groups.

**Figure 4:**
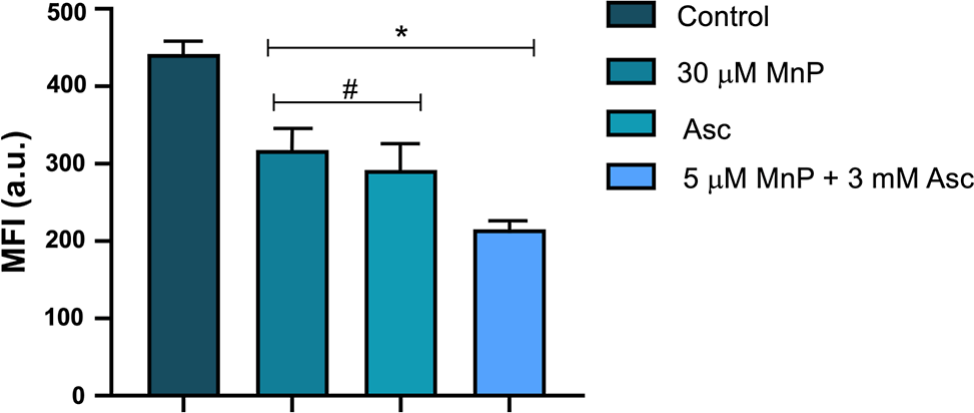
Evaluation of ΔΨm in promastigote forms of *L. amazonensis*. MnP: MnP ethyl. The concentration of Asc alone was 3 mM. *Groups statistically significant when compared to the control or ^#^ to the MnP ethyl + Asc group (*p* < 0.05). Data are presented as mean ± standard error (SE). a.u. = arbitrary units. Experiments were performed in duplicate across three independent assays.

Lastly, the combination of 5 μM MnP ethyl + 3 mM Asc resulted in a more pronounced MFI decrease of about 52%, as shown in Figure 4. These findings suggest that the combined effect between the compounds contributes to ΔΨm depolarization, leading to a decline in the parasite’s energy reserves and cell proliferation.

### Cytotoxicity Assay on Mammalian Cells

Cytotoxicity assays were conducted on both Vero cells and murine peritoneal macrophages (PMs) over a 48 h period. The results demonstrated that the MnP ethyl/Asc combination did not significantly impact (*p* ≥ 0.05) on the viability of Vero cells (Figure 5). In PMs, a mild cytotoxic effect was observed, with a slight reduction in cell viability. PMs were more susceptible to treatment with 5 μM MnP ethyl + 3 mM Asc, but cell viability was still higher than 80%. These findings reinforce the therapeutic potential of the MnP/Asc system, combining higher selectivity for parasites with minimal cytotoxicity to mammalian cells.

**Figure 5:**
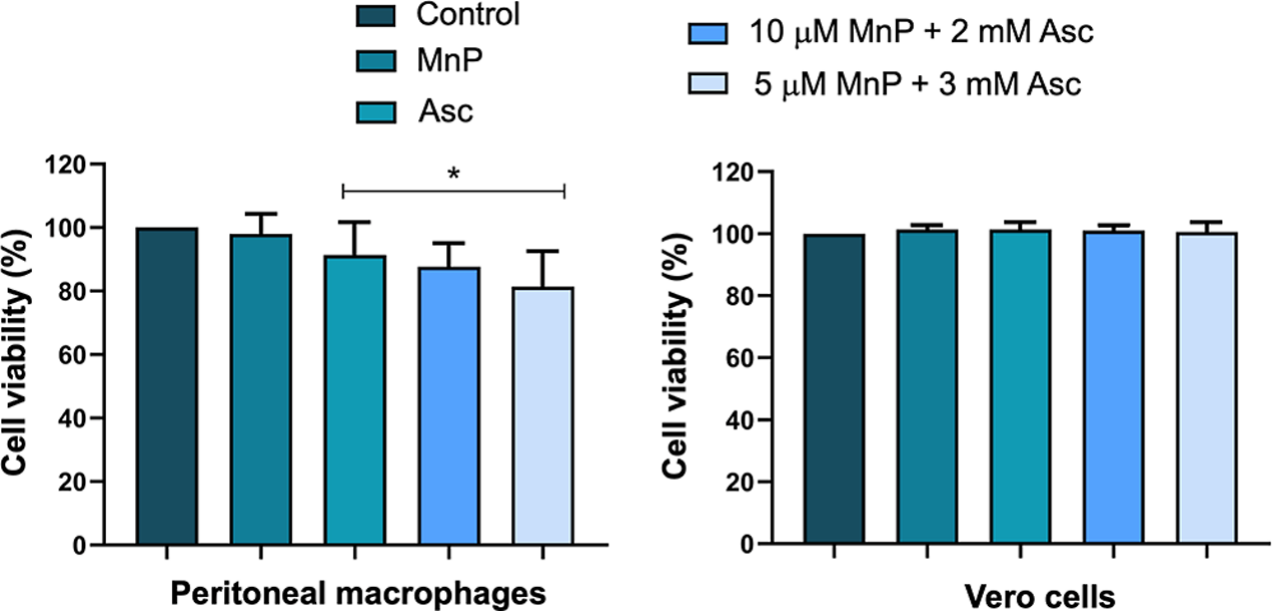
Cellular viability of mammalian cells after the treatments. Data presented as mean ± standard deviation (SD). MnP: MnP ethyl (10 μM). The concentration of Asc alone was 3 mM. *Significantly different from the control (*p* < 0.05). Experiments were performed in triplicate across three independent assays.

### Reduction of Intracellular Amastigotes by MnP Ethyl and Asc Combination

Table 1 presents the combined effect of MnP ethyl/Asc on *L. amazonensis* intracellular amastigotes. In the control group, the degree of infection (% infection) was approximately 90%, with an average of 4.3 amastigotes/macrophage and an infection index of 384. Treatment with Asc alone (1.5 mM) resulted in a slight reduction in parasite burden, decreasing the number of amastigotes *per* macrophage by 28%, the degree of infection by 8%, and the infection index by 33% compared to the control. In contrast, the combination of MnP ethyl (10 μM) and Asc (1.5 mM) significantly reduced the parasite burden, with a 42% decrease in the number of amastigotes/macrophage, a 25% reduction in the degree of infection, and a 58% reduction in the infection index after 24 h of incubation. The group treated with MnP ethyl alone showed no statistically significant differences and remained similar to the control group (data not shown).

**Table 1:** Effect of the Treatments on *L. amazonensis* Intracellular Amastigotes, 24 h Post-Incubation^a^

groups	amastigotes/macrophage	% infection	infection index
control	4.3 ± 0.8	90.0 ± 5.9	384.0 ± 73.3
5 μM MnP + 3 mM Asc + Cat	4.8 ± 0.6	85.3 ± 4.0	409.7 ± 50.9
1.5 mM Asc	3.1 ± 0.2*	82.0 ± 1.7*	255.2 ± 19.8*
10 μM MnP + 1.5 mM Asc	2.5 ± 0.8*	64.7 ± 8.2*	160.9 ± 49.3*

a The infection index was calculated following the Equation: number of amastigotes *per* macrophage × % infection. Data presented as mean ± standard deviation (SD). Control—untreated cells; MnP: MnP ethyl; Cat.: catalase 500 to 1250 units mL^–1^. Catalase was added to parasite samples in MnP ethyl/Asc treatment (evaluated at 48 h) to verify if H_2_O_2_ was involved in the cellular damage. *Significantly different from control values (*p* < 0.05). The experiments were conducted in duplicate in three independent assays.

To assess whether H_2_O_2_ was involved in the elimination of intracellular amastigotes, catalase was added to the culture medium under the same concentration and incubation conditions used for promastigotes (48 h). The presence of catalase (5 μM MnP ethyl + 3 mM Asc + Cat) resulted in a similar number of amastigotes/macrophage and degree of infection, and *ca.* 7% increase in the infection index compared to the untreated control. Thus, the addition of catalase led to a recovery in parasite proliferation, even surpassing the levels observed in the untreated control group. These findings indicate that ROS production, particularly H_2_O_2_, contributes to the effect of the MnP ethyl/Asc treatment against intracellular *L. amazonensis* amastigotes, reinforcing the potential of this combination as an antileishmanial therapeutic strategy.

## Discussion

Studies on the MnP/Asc combination in cancer cell lines have demonstrated that MnPs induce cytotoxic effects against tumor cells only in the presence of Asc. Cationic Mn­(III) ortho *N*-substituted pyridylporphyrins can act as superoxide dismutase (SOD) mimetics, designed to exert an antioxidant action by catalyzing the dismutation of O_2_^•–^ through the redox cycling between Mn­(III)P and Mn­(II)­P. In this process, Mn­(III)P reacts with O_2_^•–^ to form Mn­(II)­P, while Mn­(II)­P can be oxidized by O_2_, regenerating Mn­(III)P and producing O_2_^•–^.^13^ However, in the presence of reducing agents like Asc, MnPs switch to a pro-oxidant role. Asc reduces Mn­(III)P to Mn­(II)­P, which then reacts with molecular oxygen, amplifying O_2_^•–^ production that subsequently dismutates to H_2_O_2_.^16,17,38^ This catalytic mechanism, known as Asc autoxidation mediated by MnPs, explains why MnPs switch from an antioxidant to a pro-oxidant role under these conditions. Thus, although MnPs were originally designed as SOD mimetics, in the presence of Asc they predominantly act as pro-oxidant catalysts (Figure 6).^16,17,38^ Importantly, H_2_O_2_ is a relatively stable and highly diffusible ROS, capable of crossing membranes and promoting oxidative stress both intra- and extracellularly, which leads to cancer cell death.^15–18,38,44^

**Figure 6:**
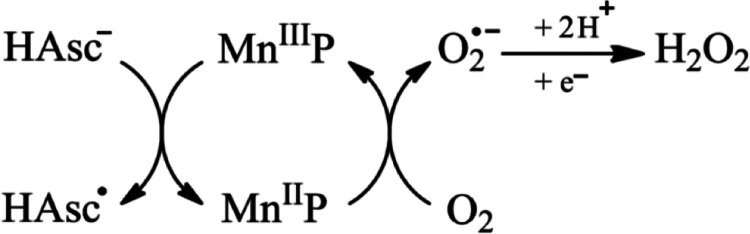
Interaction of MnP and Asc producing H_2_O_2_, adapted from.^46^

Like in cancer cells, similar effects were observed in this study with parasites: the combination of MnP ethyl and Asc significantly reduced *Leishmania* growth potentially due to oxidative damage, while MnP ethyl alone had no significant impact. This oxidative damage is supported by our results from the DCFH-DA assay, which revealed a considerable increase in ROS production following MnP ethyl/Asc treatment, indicating that the combination exerts a pro-oxidant effect on *Leishmania* cells. Moreover, the addition of catalase—an enzyme that scavenges H_2_O_2_—rescued parasite growth, suggesting that H_2_O_2_ is a key mediator of the cytotoxicity induced by the MnP ethyl/Asc combination. This mechanism corroborates the findings in cancer cells, where MnP/Asc cytotoxicity is driven by H_2_O_2_ production and accumulation.^17^

The positive charge of MnP ethyl likely facilitates its binding to negatively charged regions of the parasite’s surface, potentially leading to oxidative damage—induced by diffusible H_2_O_2_—to the plasma membrane and critical organelles following its interaction with Asc. ΔΨm results obtained after MnP ethyl/Asc treatment suggested an involvement of intracellular ROS, as the observed ΔΨm depolarization may result from an intracellular oxidative imbalance. Since catalase is generally unable to cross the cell membrane,^47^ its ability to reduce the effects of the treatment may be explained by its gradual interaction with extracellular diffusible H_2_O_2_ produced during the 48 h incubation period. Further investigations could provide more insight into these processes.

An important aspect of *Leishmania* biology is the variability in susceptibility among different species, even when exposed to the same treatment protocol.^48^ This was observed in our study, where *L. amazonensis* was the most susceptible to MnP ethyl/Asc treatment. Differences in susceptibility may be explained by variations in intracellular iron levels, which influence the Fenton reaction and OH^•^ generation.^49^ H_2_O_2_ toxicity in parasites may be associated with the Fenton reaction, which produces highly reactive OH^•^ that damage cellular components such as lipids and proteins.^50^ Previous studies have shown that iron chelation can increase resistance to H_2_O_2_-induced toxicity,^51^ reinforcing the idea that differences in iron metabolism (intracellular uptake and concentration) may underlie species-specific responses to redox-active treatments.^52,53^

In addition, trypanothione, a key antioxidant in *Leishmania*, varies among species, further influencing parasite susceptibility to oxidative stress.^24^ Trypanothione plays a crucial role in maintaining intracellular redox balance, protecting the parasite from ROS.^24,54^ In this way, if H_2_O_2_ or other ROS are present in concentrations higher than what the cellular antioxidant system can neutralize, these reactive species can interact with cellular components, such as membrane lipids and proteins. This can result in damage and, eventually, cell death.^55^

Interestingly, despite the observed reduction of *L. amazonensis* proliferation and ΔΨm depolarization by approximately 30% after treatment with 3 mM Asc, no significant increase in ROS production was detected by DCFH-DA assay. These differences observed may suggest that the antiparasitic effect of millimolar concentrations of Asc is not solely due to diffusible ROS generation but possibly involves localized oxidative stress mechanisms or intracellular metabolic disruptions. Asc at millimolar concentrations can have a pro-oxidant effect through reactions, such as Fenton chemistry in the presence of traces of heavy metal ions in the parasite cytoplasm, generating highly reactive OH^•^ within cells.^56^ The OH^•^ can cause effects on mitochondrion or interfere with other critical cellular components without contributing to measurable ROS levels in the extracellular environment.

The combination of 5 μM MnP ethyl + 3 mM Asc resulted in a more pronounced decrease in MFI (about 52% compared to untreated control), indicating a high impact on ΔΨm. This substantial depolarization suggests that the combination MnP ethyl/Asc exerts an effect on mitochondrial function, further disrupting the parasite energy production and contributing to the decline in cellular proliferation. These findings underscore the mitochondrion as a critical target of the MnP ethyl/Asc combination and reinforce the potential of this redox-active treatment to combat *Leishmania* spp. through mitochondrial destabilization. Unlike mammalian cells, *Leishmania* spp. have only a single mitochondrion. As a result, any damage caused by ROS generation to this single mitochondrion is likely to be more detrimental to this protozoon.^57^

In assays with intracellular *L. amazonensis* amastigote forms, a significant reduction in parasite load upon MnP ethyl/Asc combined treatment was observed. This suggests that this combination effectively disrupts *L. amazonensis* survival even within macrophages, where amastigotes reside. Furthermore, the presence of catalase led to a recovery in amastigote proliferation. This effect suggests that catalase may protect amastigotes by scavenging ROS generated by the MnP ethyl/Asc treatment, and reducing the impact of ROS produced by the macrophage itself, thereby contributing to parasite survival. These findings indicate that ROS production, particularly H_2_O_2,_ plays a significant role in the action of the MnP ethyl/Asc combination against *L. amazonensis*, including its intracellular forms. We believe that the interplay of MnP ethyl, Asc, H_2_O_2_, and catalase in this assay is likely similar to the one previously discussed for promastigotes.

Additionally, in assays with *L. amazonensis* amastigote forms within host cells, the Asc concentration was reduced to 1.5 mM, as infected PMs were found to be more susceptible to Asc alone. This may have occurred because infected PMs are under high oxidative stress with compromised antioxidant defenses, which could make them more vulnerable to the pro-oxidant effects of Asc. In such conditions, high concentrations of Asc can shift from acting as an antioxidant to exerting pro-oxidant activity. This effect is especially observed in environments with limited antioxidant capacity, leading to increased ROS production and potential cellular damage.^20,58^ This pro-oxidant effect has been described in tumor cells under oxidative stress, where exposure to millimolar concentrations of Asc promotes the generation of substantial amounts of ROS, enhancing cellular vulnerability.^20^ Moreover, excessive doses of Asc can cause adverse effects *in vivo*.^59,60^ Thus, one of the advantages of the MnP ethyl/Asc combination is its ability to achieve effective activity against cells with limited antioxidant capacity while using lower doses of Asc, with MnP ethyl mediating and amplifying ROS generation to enhance cytotoxicity.

As shown in Figure 5, Asc alone did not have a noteworthy impact in the viability of healthy PMs. Moreover, results indicated that MnP ethyl/Asc association exhibited minimal cytotoxicity for mammalian cells. A small variation in cell viability was observed only in PMs. It could be justified by the fact that these cells come from a primary culture, which is usually more susceptible to treatments compared to other immortalized cell lines, or even other immortalized macrophage lineages.^8,61^ This is consistent with previous reports that MnP ethyl has negligible toxicity in mammalian cells, including its evaluation in clinical trials.^37,42,43^

In tumor tissues, ROS accumulation is higher compared to healthy cells, partly due to reduced activity of antioxidant defense systems.^62^ Enzymes such as catalase, glutathione peroxidases, and peroxiredoxins, which normally detoxify H_2_O_2_, are less active in cancer cells.^43^ Similarly, *Leishmania* species possess antioxidant defense mechanisms, but their system differs significantly from that of mammals. Unlike mammalian cells, these parasites lack key enzymes, such as catalase and selenium-dependent glutathione peroxidases.^23,24^ Instead, their antioxidant defense relies on the trypanothione/tryparedoxin system, which, although specialized, operates with a more simplified mechanism. Tryparedoxin peroxidase, a key enzyme in this system, has been reported to function 10 to 100 times slower than mammalian glutathione peroxidases, contributing to the parasite’s increased susceptibility to oxidative stress.^23–25^ While this system is generally effective in protecting *Leishmania* species against endogenous oxidative challenges, it is less efficient in countering external pro-oxidant agents. When exposed to high levels of oxidative stress induced by treatments, the trypanothione-dependent system can become overwhelmed, leading to increased parasite susceptibility.^23–25,63^

Thus, the MnP ethyl/Asc combined treatment, which efficiently generates ROS (particularly H_2_O_2_), leverages on the inherent susceptibility of *Leishmania* to oxidative stress. Therefore, this redox-based therapeutic approach offers a novel and promising strategy that deserves further investigation for the treatment of leishmaniasis.

## Conclusion

This study demonstrates that the MnP ethyl/Asc combined treatment exerts significant effects in inhibiting the growth of *Leishmania* spp. promastigotes, particularly *L. amazonensis*, indicating its potential as an antiparasitic agent. The MnP ethyl/Asc combined treatment also resulted in significant depolarization of the ΔΨm of *L. amazonensis* promastigotes, suggesting a direct impact on the parasite energy reserves. Minimal toxicity was observed in mammalian cells. Different susceptibilities to the MnP ethyl + Asc combination were observed among the *Leishmania* species analyzed, encouraging further studies to investigate this behavior, which is relevant for developing effective therapeutic strategies against leishmaniasis. The MnP ethyl/Asc combination was also promising against amastigote forms. Moreover, the effect of MnP ethyl/Asc on promastigote and amastigote forms can be attributed to the production and accumulation of H_2_O_2_ as the primary mediators of the cytotoxicity induced by the redox-active treatment. This redox-active treatment has proven to be a promising and innovative approach, opening new perspectives for the development of effective and less toxic antiparasitic therapies, encouraging its evaluation in preclinical models.

## Experimental Section

### Mn­(III) Porphyrin

MnTE-2-PyP^5+^ porphyrin was synthesized in three steps: free base synthesis, its alkylation and then metalation. Briefly, H_2_T-2-PyP was prepared by condensing pyrrole and 2-pyridinecarboxaldehyde in acetic acid (solvent/catalyst) at 100 °C,^64^ using an adaptation of the Adler et al. (1964)^65^ method and purified according to a procedure described by Hambright et al. (1985).^66^ H_2_T-2-PyP was alkylated with ethyl tosylate (EtOTs), resulting in the cationic porphyrin H_2_TE-2-PyP^4+^, which was metalated using MnCl_2_·4H_2_O in an aqueous NaOH solution (pH 12.5).^67^ The resulting MnTE-2-PyP^5+^, was isolated in its chloride form (MnTE-2-PyPCl_5_) showed chromatographic, spectroscopic, thermal, and electrochemical characteristics identical to those described in the literature for MnP ethyl samples of quality for preclinical mechanistic/therapeutic purposes^33,67–70^ The MnTE-2-PyP^5+^ stock solution was prepared in H_2_O and the concentration on the order of 1.0 mM was determined by UV–vis spectroscopy using the published molar absorptivity: ε_454.0 nm_ = 138,038 M^–1^ cm^–1^ ^68^.

### Parasites

The promastigote forms of *L. amazonensis* (MHOM/77BR/LTB0016), *L. braziliensis* (MHOM/BR/1975/M2903), and *L. chagasi* (MHOM/BR/BH46) were cultivated in Schneider medium (Gibco), supplemented with 10% heat-inactivated fetal bovine serum (FBS–Gibco), 100 IU mL^–1^ penicillin, and 100 μg mL^–1^ streptomycin (Gibco). Cells were maintained at 26 °C and used during the beginning of the stationary phase of growth.

Intracellular amastigotes of *L. amazonensis* were obtained by infecting PMs with promastigotes. The PMs were isolated from peritoneal exudate cells of healthy female BALB/c mice (6–8 weeks old) and collected in 5–7 mL of ice-cold RPMI-1640 medium (Sigma-Aldrich). For the infection, PMs (5 × 10^5^ cells/well) were seeded in 24-well plates containing round coverslips (13 mm) at the bottom, allowed to adhere overnight, and then infected with promastigotes at a 10:1 ratio (promastigotes/macrophages). The cells were maintained in RPMI-1640 medium supplemented with 10% heat-inactivated FBS at 37 °C with 5% CO_2_ for 14 h. The infection was confirmed by light microscopy prior to treatments.

### Application of MnP/Asc and Assessment of Effects on Parasites

Samples of *Leishmania* spp. (promastigotes forms) were initially added in microtubes, 500 μL of the parasite suspension (1 × 10^7^ cells mL^–1^) + 500 μL of the system to be tested and then incubated for up to 48 h (in a 1:1 volume proportion). The following conditions were evaluated: (1) Control—incubation with culture medium; (2) MnP—incubation with MnP ethyl only; (3) Asc—incubation with sodium ascorbate (Sigma-Aldrich) only; (4) MnP/Asc—incubation with MnP ethyl and Asc (combined treatment). Concentrations of 5 and 10 μM of MnP ethyl and 2 and 3 mM of Asc were evaluated. These concentrations were based on studies that used this association in the context of cancer.^16,44,71^ The experiments were conducted in duplicate in at least three independent assays. After the treatments described above, the effect of MnP ethyl/Asc application on the *Leishmania* spp. was evaluated by counting the parasites using a Neubauer chamber under light microscopy.

For experiments with intracellular amastigotes, 500 μL of each system was added to 24-well plates containing macrophages infected with *L. amazonensis* and incubated for 24 h. The assay followed the same group setup used for promastigote forms, employing 1.5 mM Asc and/or 10 μM MnP ethyl. After each treatment, the cells were stained using a panoptic method according to the manufacturer’s instructions (Laborclin). The number of intracellular amastigotes/macrophage and the % of infected macrophages were quantified by analyzing 100 randomly selected macrophages per coverslip using light microscopy. The experiments were conducted in duplicate in three independent assays. The infection index was calculated by multiplying the number of amastigotes per macrophage by the % of infected macrophages. The % infection was determined as the number of infected macrophages divided by the total number of macrophages multiplied by 100.

### Investigation of Reactive Oxygen Species

The investigation into ROS generation focused on *L. amazonensis* as it showed the most significant decrease in parasite proliferation after treatment with the redox-active MnP ethyl/Asc combination.

ROS production was assessed using the DCFH-DA (Sigma-Aldrich), which is mainly oxidized by H_2_O_2_ in the presence of peroxidases, but can also react with other reactive species such as OH^•^ and ONOO^–^, although with lower specificity.^72^ For the DCFH-DA assay, promastigotes (1 × 10^6^ cells/well) were placed in black 96-well microplates and subjected to the treatments. Subsequently, 10 μM DCFH-DA was added to each well for 45 min. Fluorescence was measured over 12 h using a spectrophotometer (Spectramax M4, Molecular Devices) at λ_exc_ = 485 nm and λ_em_ = 535 nm. Experiments were performed in triplicate across two independent assays.

Bovine liver catalase (known as an H_2_O_2_ scavenger; Sigma-Aldrich) was added to the parasite culture medium at a final concentration ranging from 500 to 1250 units mL^–1^, along with the treatment (MnP ethyl/Asc) and the cells, following the same procedure described in section: Application of MnP/Asc and assessment of effects on parasites. This assay was conducted for both the promastigote and amastigote forms of *L. amazonensis*. Experiments were performed in triplicate in at least three independent assays.

### Mitochondrial Membrane Potential Analysis

To assess the effects of the treatments on the ΔΨm of the parasite, flow cytometry assays (Accuri C6, Becton Dickinson) were performed. MFIs were determined after incubating the cells (2 × 10^6^ cells mL^–1^) with the fluorescent dye Rhodamine 123 (Sigma-Aldrich), following the protocol described by Souza et al. (2021).^7^ The experiments were carried out after 6 h of incubation with the treatments. A total of 20,000 events were analyzed, and fluorescence was collected using the FL1 filter (530/30 nm) at λ_exc_ = 488 nm. This assay was also carried out in duplicate in three independent assays on the species most susceptible to the treatment.

### Cytotoxicity Assay on Mammalian Cells

The cytotoxicity assay on mammalian cells was conducted using Vero cells (ATCC CCL-81) and PMs obtained from BALB/c mice. For that, the cells were incubated in 96-well plates, containing RPMI-1640 medium, supplemented with 10% heat-inactivated FBS, at a concentration of 1 × 10^6^ cells mL^–1^. The cells were kept overnight in an incubator at 37 °C and 5% CO_2_ to adhere to the plate. The treatment groups were the same as described in section: Application of MnP/Asc and assessment of effects on parasites. After 48 h of treatment, 0.5 mg mL^–1^ of MTT (3-[4,5-dimethylthiazol-2yl]-2,5-diphenyltetrazolium bromide; Sigma-Aldrich) was added to the wells and the plates were incubated at 37 °C and 5% CO_2_ for 4 h. This assay is based on the MTT reduction by mitochondrial enzymes of viable cells, into purple formazan crystals.^73^ The formed crystals were dissolved in dimethylsulfoxide (DMSO; Neon) and the absorbances were measured in a Spectramax M4 spectrophotometer at 570 nm. Experiments were performed in triplicate across three independent assays.

### Statistical Analysis

After analyzing the normality of the data distribution using the Shapiro–Wilk test, statistical differences between groups were evaluated using the paired Student’s test. The software GraphPad Prism version 8.0 was used for data analysis and graphical generation. Differences were considered statistically significant when *p* < 0.05.

### Ethical Standards

This study was approved by the Animal Ethics Committee of Instituto Aggeu Magalhães/Fundação Oswaldo Cruz (CEUA-FIOCRUZ N° 185/2023), and all experiments were conducted in accordance with relevant ethical guidelines and regulations. Animals from the breeding facilities of Instituto Aggeu Magalhães were housed in microisolators, provided with autoclaved water and ad libitum feed. They were maintained in climate-controlled rooms with an automated air exchange system and a 12 h light/dark cycle.

## Supplementary Material



## Notes

Includes promastigote growth inhibition after 24 h of incubation with different treatments (Figure S1) and the effect of catalase addition on the efficacy of MnP/Asc treatment against *L. amazonensis* promastigotes (Figure S2) (PDF)
