# Supplementary material for: Mn(III) Porphyrin MnTE-2-PyP5+ Associated with Ascorbate: A Redox-Active Therapeutic Strategy against Leishmaniasis
Source: ACS Infect Dis. 2025 Oct 10;11(11):3128–37. doi: 10.1021/acsinfecdis.5c00520 (PMC12624719; doi:10.1021/acsinfecdis.5c00520)
Supplement: Supplementary file 1 [file id5c00520_si_001.pdf]

SUPPORTING INFORMATION

Mn(III) Porphyrin MnTE-2-PyP^{5+} Associated with Ascorbate: A Redox-Active Therapeutic Strategy Against Leishmaniasis

Tiago H. S. Souza[a,b,§,*], Jacqueline C. Bueno-Janice[a,§,*], Letícia S. Vasconcelos[a,b], Paulo E. Cabral Filho[a], Julio S. Reboucas[c], Regina C. B. Q. Figueiredo[b,#], Adriana Fontes[a,#]

[a] Departamento de Biofísica e Radiobiologia, Universidade Federal de Pernambuco (UFPE), Recife, Pernambuco, 50670-901, Brazil.

[b] Instituto Aggeu Magalhães, Fundação Oswaldo Cruz (FIOCRUZ), Recife, Pernambuco, 50740-465, Brazil.

[c] Departamento de Química, Universidade Federal da Paraíba (UFPB), João Pessoa, Paraíba, 58051-900, Brazil.

§ These authors contributed equally to this work.

# These authors jointly supervised this work.

*Corresponding authors: Tiago H. S. Souza and J. C. Bueno-Janice, Av. Prof. Moraes Rego, S/N, Departamento de Biofísica e Radiobiologia, Centro de Biociências, UFPE, 50670-901, Recife, PE, Brazil, e-mails: tiago.henriques@ufpe.br and bueno.jacqueline@gmail.com.

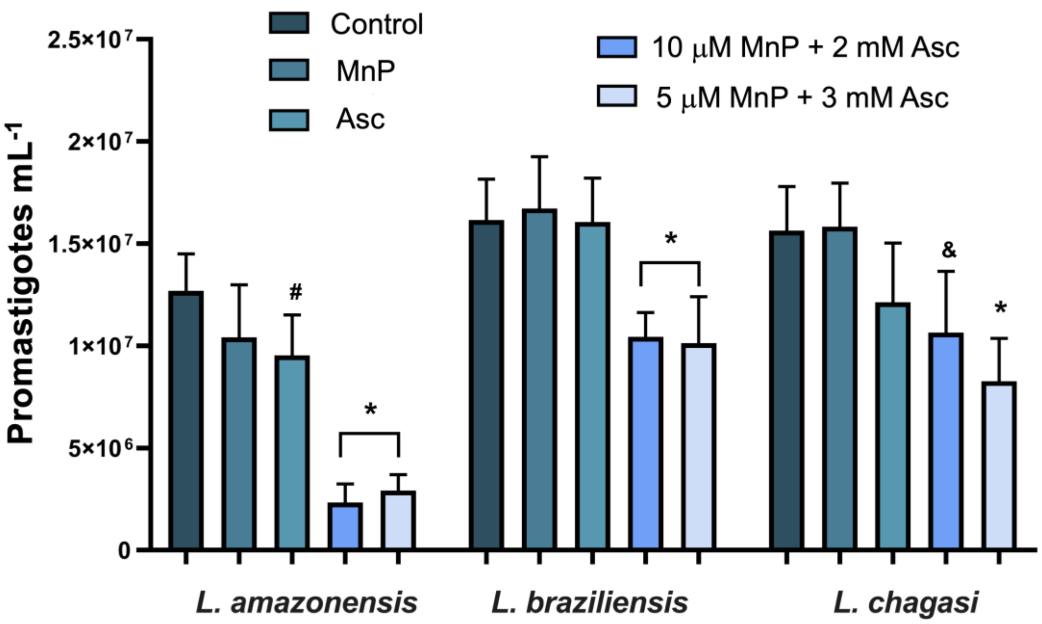

Figure S1. Effect of the treatments on the promastigote growth after 24 h of incubation. MnP: MnP ethyl (10 µM). The concentration of Asc alone was 3 mM. Data presented as mean ± standard deviation (SD). Significantly different ($p < 0.05$) from the control, MnP, and Asc groups*, between Asc and the control[#], and between the MnP/Asc groups[&]. The experiments were conducted in duplicate in at least three independent assays.

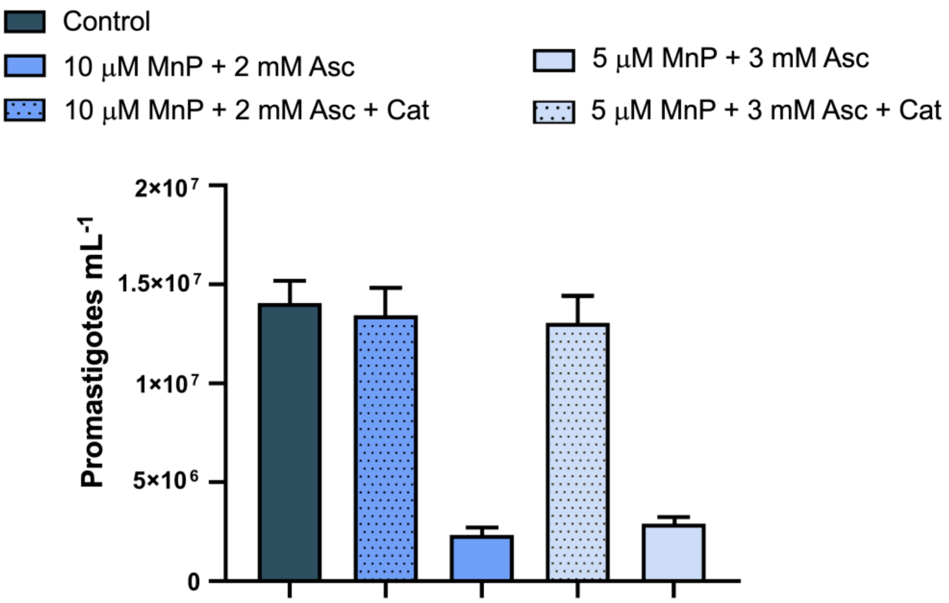

Figure S2. Reduction in the efficacy of MnP/Asc treatment on *L. amazonensis* promastigote growth after 24 h, due to the addition of catalase. Cat.: Catalase 500 to 1250 units mL^{-1}. Data presented as mean ± standard error (SE). MnP: MnP ethyl. *Significantly different from the control ($p < 0.05$). The experiments were performed in triplicate in at least three independent assays.
